# Anesthetic management of a patient with a giant goiter undergoing hip arthroplasty: A case report

**DOI:** 10.1016/j.ijscr.2025.111329

**Published:** 2025-04-21

**Authors:** Alireza Shakeri, Mohsen Shojaeian, Elham Memary

**Affiliations:** Anesthesiology Department, Imam Hossein Hospital, Shahid Beheshti University of Medical Sciences, Tehran, Iran; Anesthesiology Research Center, Shahid Beheshti University of Medical Sciences, Tehran, Iran

**Keywords:** Airway obstruction, Anesthesia, spinal, Goiter, Thyroid storm, Hip arthroplasty

## Abstract

**Introduction:**

This case report outlines the anesthetic management of a 73-year-old male with a giant multinodular goiter and uncontrolled hyperthyroidism undergoing hip arthroplasty. It highlights the challenges of balancing airway safety, endocrine stability, and surgical urgency in patients with anatomically complex goiters, emphasizing the role of multidisciplinary collaboration and spinal anesthesia as an alternative to high-risk general anesthesia.

**Case presentation:**

The patient, refusing thyroidectomy and tracheostomy, presented with a displaced femoral neck fracture and severe tracheal narrowing. Preoperative optimization included methimazole and potassium iodide to stabilize thyroid function. Spinal anesthesia using hyperbaric bupivacaine, dexmedetomidine, and fentanyl achieved a T8 sensory block, enabling uneventful cemented bipolar hemiarthroplasty. Intraoperative hemodynamics remained stable, with no sedation or airway intervention required.

**Discussion:**

Spinal anesthesia circumvented airway manipulation risks, while adjuncts prolonged analgesia without respiratory compromise. The multidisciplinary approach addressed conflicting priorities: endocrine stabilization, surgical urgency, and airway safety. Postoperative care adhered to Enhanced Recovery After Surgery (ERAS) principles, with early mobilization and non-opioid analgesia. The absence of thyroid storm or complications validated the protocol.

**Conclusion:**

This case demonstrates spinal anesthesia's efficacy in patients with giant goiters undergoing non-thyroid surgery, particularly when airway risks preclude general anesthesia. Success relied on interdisciplinary collaboration, preoperative optimization, and tailored pharmacology. Future research should explore standardized protocols for non-compliant patients and optimal adjunctive drug regimens in spinal anesthesia for high-risk populations.

## Highlights

• Successful spinal anesthesia for a giant goiter patient undergoing hip arthroplasty
• Multidisciplinary planning ensured airway safety and favorable surgical outcomes.
• Dexmedetomidine and fentanyl prolonged spinal block duration with stable hemodynamics.
• Avoidance of general anesthesia reduced airway manipulation risks.
• Thorough preoperative evaluation prevented airway-related complications.

## 1 Introduction

Goiters, characterized by abnormal enlargement of the thyroid gland, are a global health concern with a prevalence of 4–7 % in iodine-sufficient regions and up to 15–30 % in iodine-deficient areas [1,2]. While most goiters are asymptomatic, large goiters (defined as thyroid glands exceeding 80 g in weight or causing compressive symptoms) account for 5–15 % of cases and are associated with significant morbidity due to tracheal compression, dysphagia, and vascular displacement [3]. Notably, patients with large goiters frequently require non-thyroid surgeries—such as orthopedic, abdominal, or cardiovascular procedures—due to age-related comorbidities or trauma. For instance, a retrospective study of 1200 patients with giant goiters found that 22 % underwent non-thyroid surgeries within a 5-year period, highlighting the clinical relevance of anesthetic strategies tailored to this population [4].

This case report describes the anesthetic management of a 73-year-old male with a giant multinodular goiter undergoing hip arthroplasty. The case underscores the challenges of balancing airway safety, endocrine stability, and surgical urgency in patients with anatomically complex goiters. By integrating multidisciplinary planning and spinal anesthesia, this report contributes to the growing literature on optimizing perioperative care for high-risk patients with concurrent thyroid pathology and non-thyroid surgical needs.

## 2 Case presentation

### 2.1 Patient history

The patient was admitted to the hospital following a fall that caused a same-level hip fracture. He had been hospitalized a year earlier because of tachycardia and palpitations. A cardiology consultation showed normal cardiac functions; however, he was diagnosed with uncontrolled hyperthyroidism causing his symptoms. The endocrinologist advised the use of medications such as methimazole, but the patient never followed through with the treatment plan. Given the size and possible complications of the goiter, both the endocrinologists and surgeons strongly recommended a thyroidectomy, but the patient refused due to his sister's death from complications of a similarly enlarged thyroid condition and subsequent thyroidectomy.

Prior to the hip fracture, the patient was fully independent in activities of daily living (ADLs) and could ambulate without assistance. He reported the ability to walk up to two blocks without dyspnea or fatigue. Following the fall, his mobility was severely restricted, rendering him bedbound and dependent on assistance for transfers. Despite the large goiter, he had no history of dyspnea on exertion or orthopnea, consistent with the absence of respiratory distress on physical examination.

### 2.2 Patient perspective

The patient, a 73-year-old male, expressed significant anxiety regarding the proposed surgical and anesthetic plans, particularly due to his sister's prior complications following thyroid surgery. He was extensively counseled by the surgical, endocrine, and anesthesia teams about the risks and benefits of spinal anesthesia versus general anesthesia, as well as the potential complications associated with his untreated hyperthyroidism and large goiter. The patient acknowledged his understanding of the risks, including airway compromise and thyroid storm, and provided informed consent for spinal anesthesia and hip arthroplasty. He also declined tracheostomy and thyroidectomy, citing personal fears rooted in his family history. Throughout the preoperative period, the patient's concerns were addressed through repeated counseling sessions, and his preferences were respected in the final decision-making process.

### 2.3 Preoperative evaluation

Preoperative hip radiographs (anteroposterior and lateral views) confirmed a displaced femoral neck fracture (Garden type IV) with comminution of the posterior cortex. A computed tomography (CT) scan of the hip further delineated the fracture pattern and ruled out acetabular involvement. The imaging findings necessitated urgent surgical intervention to prevent avascular necrosis of the femoral head.

**Fig. 1:**
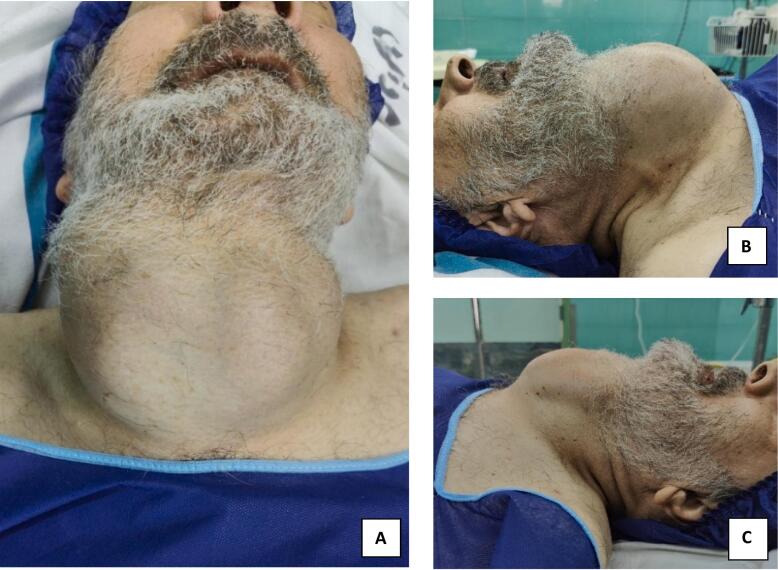
Clinical image of the patient demonstrating. A. Anterior view shows asymmetrical mass, extending below the chin and into the lower cervical area suggesting a retrosternal lesion, B right lateral view, C. left lateral view of the neck.

**Fig. 2:**
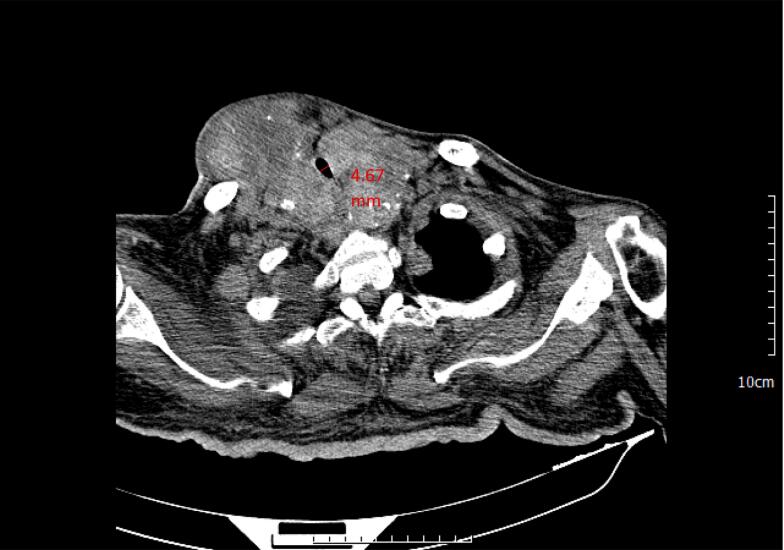
An axial cut of the CT image of the patient's neck showed the presence of a large thyroid mass lesion with heterogeneous density, causing significant asymmetry that displaced adjacent anatomical structures, including vascular and airway components (tracheal internal diameter of 4.67 mm).

The patient presented with a large goiter that was easily visible as a firm and fixed anterior neck mass (Fig. 1). A neck CT scan showed a large multinodular goiter extending retrosternally with tracheal narrowing reduced to 4.67 mm (Fig. 2). The carotid vessels and internal jugular vein were displaced posterolaterally. FNAC confirmed the diagnosis of multinodular goiter (MNG) with adenomatous nodules. Symptoms of airway obstruction included dysphagia and voice changes, but the patient denied dyspnea, even in the supine position.

### 2.4 Multidisciplinary decision-making

A consensus was reached after three joint meetings involving endocrinology, orthopedic surgery, and anesthesiology teams. The endocrinologists emphasized the imperative of thyroidectomy given the goiter's retrosternal extension and the risk of thyroid storm. However, the orthopedic team highlighted the urgency of hip arthroplasty due to the patient's immobility and pain. The anesthesiology team highlighted the anatomical risks posed by the retrosternal goiter, including tracheal compression (4.67 mm diameter) and potential airway collapse under general anesthesia. After extensive deliberation, a short delay in initiating hip arthroplasty was planned to allow for preoperative optimization of thyroid function, including achieving euthyroid status and minimizing the risk of thyroid storm. Also, modified spinal anesthesia was selected as a compromise to address both surgical urgency and airway safety. The orthopedic team committed to completing the procedure in time to prevent the need for further analgesia. The patient's refusal of tracheostomy and thyroidectomy necessitated this tailored approach.

### 2.5 Preoperative thyroid stabilization

To minimize thyroid storm risk, methimazole 5 mg twice daily and potassium iodide (Lugol's solution) 5 drops orally every 8 h were initiated three days preoperatively. Thyroid function was monitored daily, with TSH stabilizing at 0.51 μIU/mL, T4 at 6.4 μg/dL, and T3 at 1.53 ng/mL. Endocrinology clearance was contingent on maintaining euthyroid status, achieved through strict adherence to this regimen. A beta-blocker (propranolol 20 mg twice daily) was kept on standby for intraoperative tachycardia.

### 2.6 Anesthetic plan and rationale

Apart from the patient being refused tracheostomy, due to paratracheal extension of goiter, tracheostomy was also not feasible which complicated the airway management issue further. An ETT size of 3.5 was decided upon initially, estimating a narrowed tracheal lumen; however, the length of the tube was inadequate to extend down to and secure the trachea. The small size, apart from the length inadequacy of the tube, caused high airway resistance and was a cause for concern regarding ventilation and oxygenation. The inability to use flexible fiber optics due to the required ETT size further limited options, making rigid bronchoscopy the only viable alternative as a rescue option [5].

**Fig. 3:**
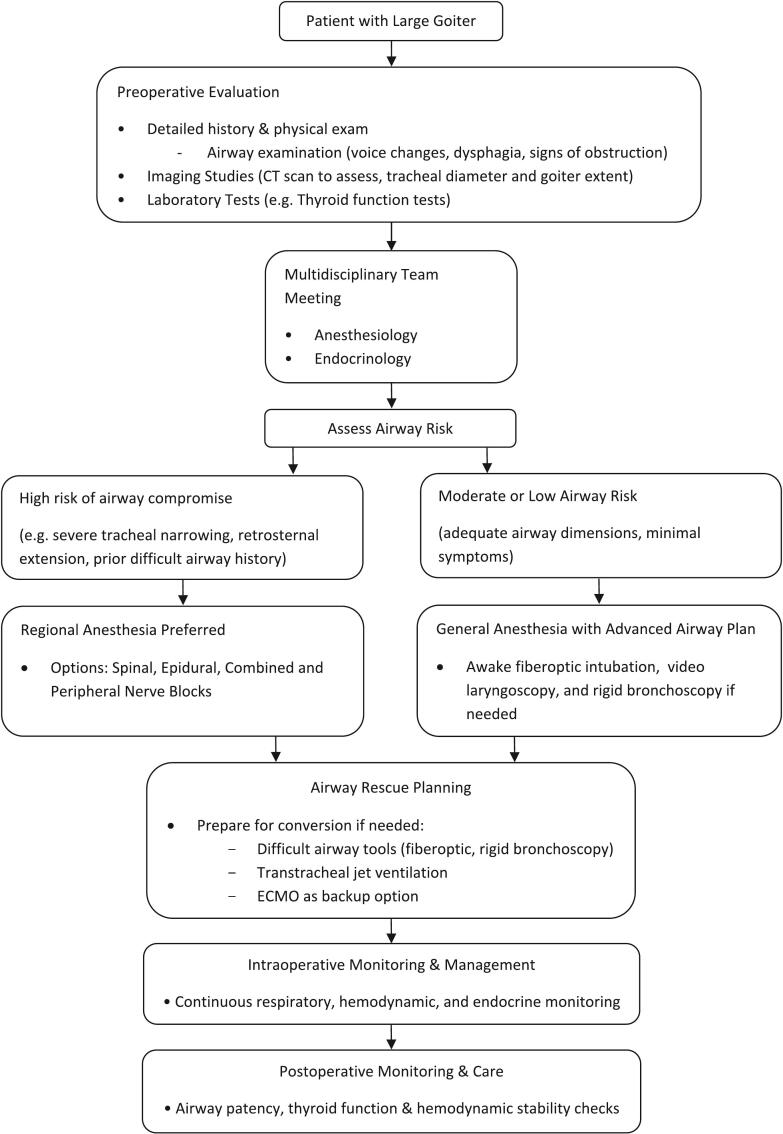
Multidisciplinary algorithm for airway management, anesthetic technique selection, and contingency planning in patients with giant goiters.

The anesthetic strategy was meticulously crafted through interdisciplinary consensus, balancing the competing priorities of surgical urgency, airway safety, and endocrine stability. General anesthesia was unanimously deemed untenable due to the patient's anatomically compromised airway (Fig. 3).

Spinal anesthesia was thus selected to circumvent airway instrumentation entirely, minimizing physiological stress and eliminating the risk of iatrogenic airway trauma [6]. The spinal anesthetic protocol incorporated hyperbaric bupivacaine (5 mg), supplemented with fentanyl (25 μg) and dexmedetomidine (5 μg). This combination was deliberately chosen to optimize block duration and quality without escalating the local anesthetic dose, thereby avoiding cephalad spread that could compromise respiratory function in a patient with preexisting tracheal narrowing. Dexmedetomidine, an α2-adrenergic agonist, synergistically potentiates the density of the block and prolonged sensory and motor blockade intended to prolong the duration of anesthesia effectively equal to that provided by an epidural, without the need for catheterization [7]. Furthermore, dexmedetomidine attenuates sympathetic hyperactivity, which is a critical consideration given the patient's hyperthyroid state [8,9]. Fentanyl enhanced analgesia by targeting spinal opioid receptors, prolonging the duration of the block, and reducing postoperative opioid requirements [10,11]. Technical adjustments were necessitated by the patient's limited mobility from his hip fracture and degenerative spinal changes; a lateral decubitus position and a 25-gauge Quincke needle at the L4-L5 interspace facilitated successful administration, achieving a T8 sensory block sufficient for surgery [12].

These were communicated to the surgical team, and the need for the surgery to be kept as short as possible was communicated in order to provide the highest level of safety to the patient. The surgical team was fully prepared in advance to carry out a surgery in quick time with a high level of momentum.

Alternative regional techniques, such as lumbar plexus block, fascia iliaca block, quadratus lumborum block, erector spinae plane block, or a combination of these blocks are recognized for their safety profile and potential for rapid recovery, and their increasing use in many surgeries as well as hip arthroplasty is well-documented as main anesthetic technique [13,14]. However, in most cases, concomitant sedation is necessary, and the risk of airway compromise may be possible. Therefore, these methods were not selected in this case despite their potential feasibility, and tailored spinal anesthesia was chosen for its reliability in providing complete surgical anesthesia, avoiding airway manipulation, and minimizing the risk of conversion to general anesthesia. Similarly, epidural anesthesia was ruled out owing to the patient's degenerative spinal anatomy, characterized by age-related spondylotic changes and limited spinal flexibility, posed significant technical barriers to safe epidural catheter placement. These anatomical constraints, coupled with the patient's limited ability to cooperate during positioning due to acute hip fracture pain and immobility, increased the risk of procedural complications such as dural puncture or incomplete analgesia.

### 2.7 Intraoperative management

The procedure was performed at Imam Hossein Hospital, a tertiary medical and educational care center with specialized facilities for orthopedic and endocrine management. The surgical team was led by an experienced orthopedic surgeon with over 15 years of expertise in hip arthroplasty, assisted by a senior resident and a scrub nurse. The anesthesiology team, comprising a consultant anesthesiologist and a resident, was responsible for spinal anesthesia administration and perioperative monitoring.

Contingency planning played a pivotal role in the anesthetic strategy. A thyroid storm protocol was activated, with propranolol (20 mg twice daily) and esmolol boluses prepared for intraoperative tachycardia, alongside cooling blankets for hyperthermia. Airway rescue measures, including rigid bronchoscopy and transtracheal jet ventilation, were prearranged but ultimately unnecessary.

The patient underwent cemented bipolar hemiarthroplasty under spinal anesthesia. A 25-gauge Quincke needle was used at the L4-L5 interspace to administer a mixture of hyperbaric bupivacaine (5 mg), fentanyl (25 μg), and dexmedetomidine (5 μg). The sensory block ascended to T8 within 10 min, providing adequate surgical anesthesia.

Blood pressure remained stable throughout the procedure, ranging between 120 and 135/70–85 mmHg, and no vasopressors were required. Heart rate averaged 75–85 beats per minute, with no episodes of tachycardia or bradycardia. Oxygen saturation was maintained at 97–99 % on 2 L per minute of supplemental oxygen delivered via nasal cannula. Continuous electrocardiographic (ECG) monitoring demonstrated sinus rhythm without arrhythmias or ischemic changes. Notably, no sedatives were administered during the operation, ensuring minimal risk of airway compromise.

The patient underwent a cemented bipolar hemiarthroplasty via a posterolateral approach. The surgical team utilized a standardized protocol for hip arthroplasty in elderly patients with fractures. After the incision, the short external rotators and posterior capsule were meticulously dissected to minimize soft tissue trauma. The fractured femoral head was excised, and the acetabulum was inspected for integrity; no degenerative changes were noted. A cemented femoral stem (size 12) with a 46 mm bipolar head was implanted after careful reaming and lavage. Intraoperative fluoroscopy confirmed proper alignment and cement mantle distribution. The posterior capsule and external rotators were repaired using non-absorbable sutures to reduce dislocation risk. The total surgical duration was 60 min, with an estimated blood loss of 300 mL. No intraoperative complications, such as cement implantation syndrome were observed.

### 2.8 Postoperative management

The patient was taken to the post-anesthesia care unit (PACU) in stable condition following the surgery. The patient remained in the PACU for 2 h, during which he was continuously monitored for hemodynamic stability, respiratory function, and signs of thyroid storm by tachycardia, hypertension, hyperthermia, and changes in mental status. The patient was further kept under observation regarding his goiter for the onset of respiratory distress and other complications. Discharge from the PACU was contingent upon meeting several criteria, including stable hemodynamics with blood pressure 110/60 mmHg and heart rate of 70 beats per minute, oxygen saturation of at least 96 % on room air, full return of motor function (Bromage score 0), adequate pain control (Visual Analog Scale score 1/10), and an Aldrete score of 10. These criteria were met at the 2-h mark, allowing for safe transfer to the ward.

Postoperative pain was managed with scheduled intravenous acetaminophen (1 g every 8 h) and ketorolac (30 mg every 12 h for 24 h), transitioning to oral acetaminophen (1 g every 6 h as needed) after 24 h. The pain was managed effectively with a combination of intravenous and oral non-opioid analgesics as the spinal block wore off. This ensured adequate pain relief while protecting the patient's airway stability in the crucial postoperative period. Thyroid function tests (TSH, T3, T4) were repeated at 6-h intervals for 24 h, with results remaining within euthyroid ranges.

Postoperative radiographs confirmed satisfactory implant positioning with no evidence of periprosthetic fracture or malalignment. The patient was mobilized on postoperative day 1 with partial weight-bearing using a walker, guided by physiotherapy. Thyroid function tests (TSH, T3, T4) remained stable throughout the hospital stay, and no signs of thyroid storm emerged. The patient was discharged on postoperative day 5 with outpatient follow-up arranged for thyroid monitoring and hip rehabilitation. At the 6-week follow-up, the hip incision had healed without infection, and the patient reported significant pain relief (VAS score: 2/10 at rest). Repeat radiographs demonstrated stable implant positioning and early signs of fracture union.

## 3 Discussion

General anesthesia in patients with large goiters poses significant risks due to anatomical distortion and tracheal compression, which can lead to difficult intubation, failed ventilation, and postoperative airway obstruction [5]. Severe tracheal narrowing increases these risks, and induction agents may exacerbate airway collapse by reducing tracheal wall rigidity, necessitating advanced techniques like rigid bronchoscopy [15]. Additionally, general anesthesia can precipitate thyroid storm in hyperthyroid patients.

This case diverges significantly from standard protocols for giant goiters, where preoperative thyroidectomy or sometimes tracheostomy is prioritized to mitigate airway risks to prevent sudden, life-threatening complications [16,17]. While literature doesn't specifically address refusal scenarios, when a patient with goiter refuses the recommended thyroidectomy and tracheostomy, the clinical team faces complex ethical and medical considerations. Alternative approaches may considered as this case underscores the viability of spinal anesthesia in high-risk, non-compliant patients, a scenario paralleled only in rare reports like Moreira et al., where multidisciplinary collaboration achieved safety without invasive airway measures where Extracorporeal Membrane Oxygenation (ECMO) as backup [18].

The adjunctive use of dexmedetomidine further distinguishes this case. Consistent with our study, some studies demonstrate its role in prolonging neuraxial blockade is recognized in obstetric, abdominal, and lower extremities surgeries [7,19]. However, its application here has dual benefits—prolonging blocked time and attenuating sympathetic hyperactivity — as utilizing intravenous dexmedetomidine was discussed in the previous literature [8,9]. The decision aligns with evidence suggesting that single-shot spinal anesthesia with adjuncts can achieve comparable efficacy to epidural techniques in select populations, particularly when anatomical or logistical challenges preclude neuraxial catheter placement [19,20]. This approach not only prevented high spinal anesthesia but also minimized opioid requirements, a critical consideration in elderly patients with comorbidities.

The study emphasizes the multidisciplinary framework essential for managing complex, high-risk patients. The preoperative stabilization of thyroid function in a non-compliant patient, combined with rigorous contingency planning for thyroid storm, offers a replicable model for cases where standard interventions are declined, filling a gap highlighted by de Mul et al. in managing non-compliant hyperthyroid patients [21]. While prior research has highlighted perioperative thyroid management [22,23], few reports detail protocols for patients refusing standard therapies. Our experience demonstrates how collaborative decision-making can reconcile patient autonomy with clinical necessity, a nuance increasingly relevant in modern patient-centered care.

This report also highlights the technical considerations for hip arthroplasty in patients with concurrent systemic comorbidities. The choice of a cemented femoral stem was deliberate, prioritizing immediate stability in an osteoporotic femur, while the posterolateral approach balanced surgical exposure with soft tissue preservation [24,25]. The absence of intraoperative complications, such as cement-related hypotension or femoral perforation, underscores the importance of meticulous surgical technique and interdisciplinary coordination. Furthermore, the patient's rapid mobilization postoperatively aligns with current ERAS (Enhanced Recovery After Surgery) protocols for hip fracture management, which emphasize early physiotherapy to reduce morbidity in elderly patients [26,27].

## 4 Conclusion

This case exemplifies the safety of spinal anesthesia in complex airway scenarios and underscores the value of integrating orthopedic surgical precision with perioperative endocrine stabilization to achieve favorable outcomes in high-risk patients. While spinal anesthesia provided the technical foundation, its success hinged on preoperative endocrine optimization, contingency planning for thyroid storm, and seamless interdisciplinary collaboration. Together, these elements transformed a potentially catastrophic scenario into a controlled intervention, offering a replicable framework for clinicians managing analogous challenges.

Future studies could explore optimal drug combinations for spinal anesthesia in different situations, particularly in patients with severe airway compromise. Also, standardized perioperative protocols for patients refusing definitive treatments warrant further investigation to balance autonomy and clinical urgency. These directions could refine perioperative strategies and improve patient care.

## CRediT authorship contribution statement

Mohsen Shojaeian contributed to the study concept and design, data collection, and interpretation.

Elham Memary was responsible for data analysis and drafting the manuscript.

Alireza Shakeri provided critical revisions and final approval of the manuscript.

All authors have read and approved the final version of the manuscript and agree to be accountable for all aspects of the work.

## Consent process

Written informed consent was obtained from the patient after a detailed discussion of the surgical and anesthetic plans, including the rationale for spinal anesthesia, potential complications, and contingency measures. The consent process was documented in the medical records, and the patient was assured of his right to withdraw consent at any time. A copy of the written consent form is available for review by the Editor-in-Chief upon request. The patient also consented to the publication of this case report and accompanying images, understanding that his identity would remain confidential.

## Ethical approval

Ethical approval was not required for this case report in accordance with the institution's guidelines.

## Guarantor

Mohsen Shojaeian accepts full responsibility for the work, had access to the data, and controlled the decision to publish this case report.

## Research registration number

Our case report does not fall under the category of a “First in Man” study; therefore, registration is not required.

## Declaration of Generative AI and AI-assisted technologies in the writing process

During the preparation of this work, the authors used DeepSeek, an AI-assisted language model, to enhance language clarity and readability, and Grammarly to ensure proper grammar usage. After using these tools, the authors reviewed and edited the content as needed and took full responsibility for the content of the publication.

## Funding

No external funding was received for this case report.

## Declaration of competing interest

The authors declare that they have no conflicts of interest to disclose.
